# Glucocorticoids Improve the Pregnancy Rate and Outcome in Women With Unexplained Positive Autoantibodies: A Systematic Review and Meta-Analysis

**DOI:** 10.3389/fmed.2022.819406

**Published:** 2022-05-11

**Authors:** Ting Li, Yilin Yuan, Huixin Liu, Qun Lu, Rong Mu

**Affiliations:** ^1^Department of Rheumatology and Immunology, Peking University Third Hospital, Beijing, China; ^2^Department of Psychiatry, Peking University Sixth Hospital, Beijing, China; ^3^Department of Clinical Epidemiology and Biostatistics, Peking University People’s Hospital, Beijing, China; ^4^Reproductive Medical Center, Peking University People’s Hospital, Beijing, China

**Keywords:** glucocorticoids, autoantibody, meta-analysis, pregnancy, outcome

## Abstract

The effect of glucocorticoid therapy on women with unexplained positive autoantibodies is under debate. This systemic review and meta-analysis were performed to evaluate whether glucocorticoid administration can improve the pregnancy outcome of this population. Relevant publications were searched from databases, and a total of seven prospective and retrospective cohort studies that investigated the effects of glucocorticoid administration on women with unexplained positive autoantibodies, were included. The outcomes of our systematic review and meta-analysis were measured in terms of risk ratios (RR) with 95% confidence intervals (CI) using fixed or random effect models. We found that glucocorticoid treatment improved the clinical pregnancy rate (RR 2.19, 95% CI 1.64–2.92) and live birth rate (RR 1.92, 95% CI 1.17–3.16), especially when glucocorticoid administration was started before pregnancy (clinical pregnancy rate: RR 2.30, 95% CI 1.58–3.34; live birth rate: RR 2.30, 95% CI 1.58–3.34). However, no effect of glucocorticoids on the miscarriage rate was found (RR 0.75, 95% CI 0.55–1.02) regardless of the time of drug administration. Our systematic review and meta-analysis support the rational use of glucocorticoids in women with unexplained positive autoantibodies.

**Systematic Review Registration:**

[www.ClinicalTrials.gov], identifier [CRD42019124442].

## Introduction

Human fertility is declining globally. Around 9% of the couples are involuntarily childless (1). Infertility or miscarriage, in a broad sense, is the result of failed establishment or maintenance of pregnancy. Although there are advancements in technology, such as *in vitro* fertilization-embryo transfer (IVF-ET), the cumulative live birth rate after six cycles is about 51–72%, and this number will drop to 23–42% among patients who are 40 years of age or older (2), which is a frustrating experience for both patients and clinicians. Improving pregnancy outcomes is of great importance. Many factors can lead to a miscarriage, such as the age during the pregnancy, maternal reproductive system malformation, and embryo chromosome aberrations (3). However, maternal immune tolerance to the placenta and the fetus is one of the key factors in establishing and maintaining pregnancy. Immunological abnormalities were reported to account for 27.88% of the fetal loss, including overproduction of autoantibodies and proinflammatory cytokines and an imbalance of immune cells (4, 5). Women with autoantibody positivity can be divided into two groups based on whether they are diagnosed with autoimmune diseases. The chance of live birth was significantly reduced in women with rheumatoid arthritis receiving ART treatment relative to women without rheumatoid arthritis (6). Antiphospholipid syndrome accounts for ∼3.2% of the recurrent pregnancy loss in the first trimester (7). However, these diseases only account for a small proportion of all immune factors affecting obstetrics. Regarding more frequent cases, autoantibody-positive women with recurrent fetal loss or a history of recurrent implantation failure who cannot be classified into any known autoimmune disease are insufficient. It remains marginalized in terms of improving the pregnancy outcome of this population.

For infertile women with unexplained positive autoantibodies, empirical treatment is a mainstay possession. Several therapies have been proposed in daily practice (8), among which glucocorticoids are a group of classic drugs for autoimmune diseases. However, the effect of glucocorticoids in improving pregnancy outcomes in women with unexplained positive autoantibodies is controversial. Furthermore, disputes about glucocorticoid treatment remain in matters of drug selection, initial treatment time, the dosage of glucocorticoids, and period of treatment. It is necessary to determine whether glucocorticoid treatment is beneficial to pregnancy outcomes in women with unexplained positive autoantibodies, as the treatment regimen is quite arbitrary and confusing in daily practice. Therefore, we reviewed the clinical research content and performed a systematic review and meta-analysis on the treatment effect of glucocorticoids in this population to provide evidence for rational drug use in women with unexplained positive autoantibodies.

## Materials and Methods

### Search Strategy and Selection Criteria

This study was registered with PROSPERO (registration number: CRD42019124442) and was conducted and reported according to the Preferred Reporting Items for Systematic Review and Meta-Analysis (PRISMA) statement (9). We conducted a systematic search both in English and Chinese in PubMed, Embase, EBSCO, and the Cochrane Central Register of Controlled Trials. For a comprehensive literature search, a string made up of relevant keywords was utilized (“glucocorticoids” or “steroid” or “prednisone” or “prednisolone” or “methylprednisolone” or “dexamethasone”) and (“recurrent fetal loss” or “recurrent pregnancy loss” or “recurrent spontaneous miscarriage” or “infertility” or “assisted reproductive technique” or “IVF-ET” or “recurrent implantation failure”) and (“autoimmune” or “autoantibody”). The time of publication was not limited.

Two independent reviewers (Yilin Yuan and Ting Li) reviewed the titles and abstracts for basic relevance, followed by a full-text examination using the following inclusion and exclusion criteria. Only randomized controlled trials (RCTs) and cohort studies investigating the use of glucocorticoids in women with unexplained positive autoantibodies undergoing pregnancy were assessed based on full text. Any discordant findings between the two independent reviewers were adjudicated by a third reviewer.

The inclusion criteria were as follows: unexplained recurrent fetal loss or infertility; autoantibody positivity but not meeting any classification criteria for autoimmune diseases; intervention group treated with glucocorticoids; and control group given placebo or untreated.

The exclusion criteria included repeated data, diagnosis with a specific autoimmune disease, lack of positive autoantibodies, lack of glucocorticoid administration, and risk of confounding factors such as thyroid function anomalies.

### Quality Assessment

RCTs were quality assessed based on the criteria outlined in the Cochrane Handbook for Systematic Reviews of Interventions (10). Retrospective cohort studies were quality assessed based on the New Castle-Ottawa Quality Assessment Scale-Cohort Studies. Two authors independently assessed every paper.

### Data Extraction and Analysis

The following data were extracted: the number of enrolled patients, the status of autoantibodies, type of glucocorticoids, initial glucocorticoid treatment time, the dosage of glucocorticoids, period of treatment, combination therapy, number of clinical pregnancies, live births, and miscarriages (per pregnancy).

The primary outcome of our study was the relative risk (RR) of establishing clinical pregnancy comparing glucocorticoid-treated and non-glucocorticoid-treated groups.

The secondary outcomes in our study were (1) RR of live birth rate (per couple) comparing glucocorticoid-treated and non-glucocorticoid-treated groups and (2) RR of the miscarriage rate (per pregnancy) comparing glucocorticoid-treated and non-glucocorticoid-treated groups.

We examined the heterogeneity (variations) between the results of different studies by checking the results of the chi-squared Cochran’s Q statistic and I^2^ statistics. I^2^ > 50% represents statistical significance for heterogeneity, and random-effects models were used if heterogeneity was present. All analyses were conducted by the Cochrane Collaboration Review Manager 5.3 software package.

## Results

**FIGURE 1:**
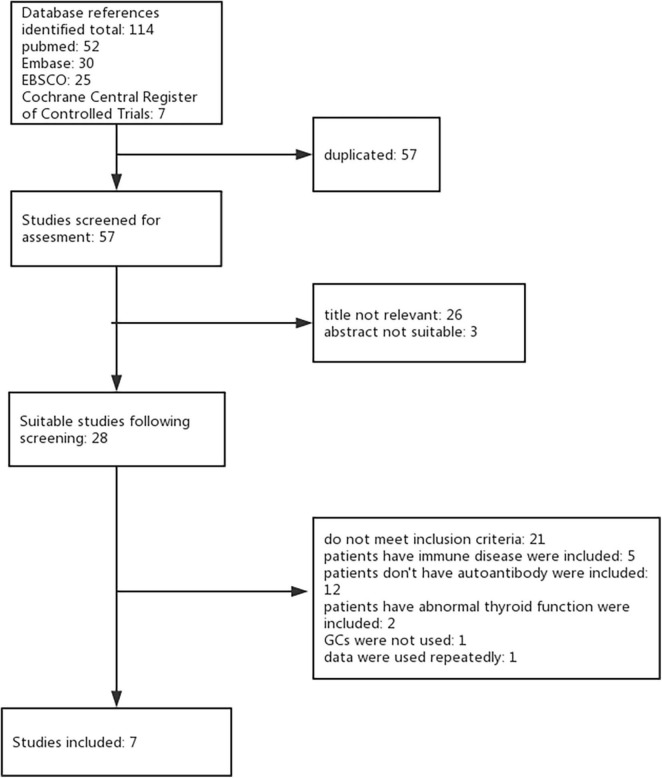
The process of finding studies.

Using the keywords and databases listed above, seven studies were identified for inclusion. During the initial search, we identified a total of 114 studies by searching strategy in all four databases. After eliminating 57 duplicate records and 29 studies not relevant to our research question, 28 studies remained suitable for subsequent screening. Among these, we excluded 21 studies (11–31) (see Supplementary Table 1 for studies excluded and reason for exclusion). Seven studies met the inclusion criteria with no reason for exclusion providing data comparing peri-implantation glucocorticoids versus placebo (or untreated). Full agreement existed between the two review authors concerning inclusion or exclusion trials (Figure 1).

Among the seven studies identified, five studies were parallel-design RCTs, and two studies were cohort studies. All were single-center studies. The risk of bias is listed in Supplementary Tables 2, 3.

### Participants

A total of 681 participants were included from seven independent studies. Among these studies, one study further divided its participants into subgroups with positive ANA or APL (32) (Table 1). Three studies included women who were positive for one of the autoantibodies, including antinuclear antibody (ANA), anti-DNA (anti-dsDNA by Geva et al. anti-ssDNA and anti-dsDNA by Ando et al. and Laskin et al.), anticardiolipin antibody (ACL), lupus anticoagulant (LAC), and antilymphocyte antibody (32–34); two studies included only women who were positive for ANA (35, 36); and one study included only women who were positive for antiphospholipid antibody (APL). (37) One study included women who had thyroid peroxidase antibodies (TPO-Abs) with normal thyroid function (38).

**TABLE 1:** Characteristics of participants in the included studies.

Study	Age	Indication for treatment	Autoantibody profile	GCs use	Adjuvant therapy
Fan, 2016 (36)	31.1 ± 4.2	IVF-ET failure	Positive for ANA	Prednisone: 10 mg/d Started 3 months before COH Stopped if confirmation of clinical pregnancy	Aspirin: 100 mg/d Administered simultaneously with GCs
Turi, 2010 (38)	34.4 ± 3.8	Infertility	Positive for antithyroid antibody	Prednisone: 10 mg/d × 1 week, 5 mg/d × 1 week, 2.5 mg/d × 1 week, and 2.5 mg/d × 3 times Started 4 weeks before IUI Stopped just before IUI	
Geva, 1998 (33)	n.d.	IVF-ET failure	Positive for ANA, anti-ds DNA, ACL or LAC	Prednisone: 10 mg/d Started 4 weeks before induction of ovulation Stopped at 18^th^ week of gestation, IVF-ET failure or fetal loss	Aspirin: 100 mg/d Started simultaneously with GCs Stopped 6 weeks’ postpartum, IVF-ET failure or fetal loss
Laskin, 1997 (34)	3 ± 3.8	RPL	Positive for ANA, anti-ds DNA, ACL, LAC or anti-lymphocyte	Prednisone: 0.8 mg/kg/d × 4 weeks (maximum, 60 mg), 0.5 mg/kg/d (maximum, 40 mg) Started since confirmation of pregnancy Stopped at delivery or fetal loss	Aspirin: 100 mg/d Started simultaneously with GCs Stopped at 36^th^ week of gestation or shortly before delivery
Ando, 1996^†^ (32)	32.8 ± 3.4	n.d.	Positive for ANA, anti-DNA or LAC	Prednisolone 5 mg/d Or dexamethasone 0.5 mg/day, changed into prednisolone after confirmation of pregnancy Started with IVF cycle Stopped according to autoantibody titers	Aspirin: 81 mg/d Administered simultaneously with GCs
Zhu, 2013 (35)	32.33 ± 4.25	Infertility	Positive for ANA	Prednisone: 10 mg/d Up to 3 months before IVF/ICSI cycle	Aspirin: 100 mg/d Administered simultaneously with GCs
Ying, 2012 (37)	32.1 ± 4.0	Infertility	Positive for ACL	Methylprednisolone: 8 mg/d Up to 3 months before IVF cycle	Aspirin: 50 mg/d Administered simultaneously with GCs

*RPL = recurrent pregnancy loss, ANA = antinuclear antibody, ACL = anticardiolipin antibody, LAC = lupus anticoagulant, APL = antiphospholipid antibody, COH = controlled ovarian hyperstimulation, IUI = intrauterine insemination, IVF-ET = in vitro fertilization-embryo transplantation, ICSI = intracytoplasmic sperm injection, n.d. = not defined.*

*^†^Further divided into subgroups according to autoantibodies, including ANA(+) and LAC(+).*

*^‡^Further divided into 2 subgroups according to autoantibodies, including ANA(+)/APL(−) and ANA(+)/APL(−).*

### Intervention

A variety of different protocols for glucocorticoid administration were used. The types of glucocorticoids were prednisone (33–36, 38), methylprednisolone (37), prednisolone (32), and dexamethasone (32). Among these studies, most of them used only one particular glucocorticoid, except that Ando et al. prescribed two types of glucocorticoids, either dexamethasone or prednisolone, with no explanation of the basis for choosing which one to give (32).

Glucocorticoids were mostly used in low doses. However, the dose schedules and length of treatment were variable (Table 1). Methylprednisolone was used at a dose of 8 mg/d by Ying et al. (37). Prednisone was used at a dose of 10 mg/d by Zhu et al., Geva et al., and Fan et al. (33, 35, 36). Turi et al. started prednisone administration at a dose of 10 mg/d in the 1st week and then gradually reduced the dosage until complete withdrawal (38). Laskin et al. prescribed prednisone 0.8 mg/kg/d for 4 weeks (maximum, 60 mg/d) from confirmation of pregnancy by ultrasonography, followed by 0.5 mg/kg/d (maximum 40 mg/d) until delivery or fetal loss (34). Ando et al. prescribed prednisolone 5 mg/d or dexamethasone 0.5 mg/d during the entire IVF cycle and changed it to prednisolone 5 mg/d after confirmation of pregnancy (32). All studies used only oral regimens.

Among the six studies involving ART, the assisted reproductive technique differed. The most assisted reproductive technology (ART) conducted was IVF-ET (32, 33, 35–37), except for one study that used IUI (38), and 1 study did not mention whether ART was used (34). In the six studies, treatment initiation was related to the ART cycle, and glucocorticoids were provided consecutively during both the luteal phase and follicular phase. Glucocorticoids were administered either up to 3 months before the IVF/ICSI cycle (35, 37) and induction of ovulation (36) or 4 weeks before induction of ovulation (33) or intrauterine insemination (IUI) (38). Ando et al. (32) administered glucocorticoids from the IVF cycle. In 1 study without ART, glucocorticoids were provided from the confirmation of pregnancy (34).

Additional aspirin 100 (33–36), 81 (32), or 50 mg/d (37) was provided as an adjuvant in most included studies except the one by Turi et al. (38).

### Outcome

There were six studies reporting the clinical pregnancy rate (32, 33, 35–38). The live birth rate was obtained from six studies (32–34, 36–38). In one study, the clinical observation stopped after the first trimester, so we classified those who did not miscarry by the end of the study as ongoing pregnancy (35). Concerning the miscarriage rate, six studies reported the total miscarriage rate (32–34, 36–38). Geva et al. and Zhu et al. reported early miscarriage as well (33, 35). Other incidences in pregnancy were also recorded in some studies, including preterm birth, intrauterine death, neonatal death, ectopic pregnancy, hypertension (HTN), and gestational diabetes mellitus (GDM) (33, 34).

### Effects of Interventions

#### Clinical Pregnancy Rate per Couple

**FIGURE 2:**
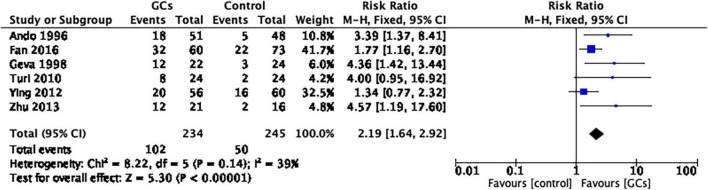
Forrest plot for comparison: glucocorticoids versus no glucocorticoids/placebo, outcome: I. I Clinical pregnancy rate per couple. Six studies reported the clinical pregnancy rate per couple. This result favors glucocorticoid use in improving the clinical pregnancy rate (RR 2.19, 95% CI 1.64 –2.92, *p* < 0.00001, six cohort studies, 479 women, I^2^ = 39%).

In six studies reporting the clinical pregnancy rate per couple, glucocorticoid use improved the clinical pregnancy rate (RR 2.19, 95% CI 1.64–2.92, *p* < 0.00001, six cohort studies, 479 women, *I*^2^ = 39%; Figure 2).

**FIGURE 3:**
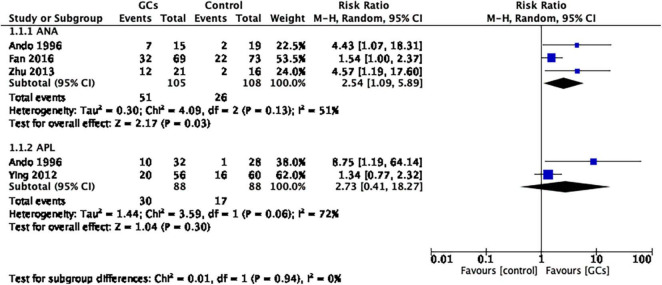
Forrest plot comparison: glucocorticoids versus no glucocorticoids/placebo, outcome: I. II Clinical pregnancy rate per couple in subgroups by autoantibody. The ANA-positive subgroup favored glucocorticoid therapy, while the APL-positive subgroup did not (ANA: RR 2.54, 95% CI 1.09 –5.89, *p* = 0.03, three cohort studies, 213 women, I^2^ = 51%; APL: RR 2.73, 95% CI 0.41–18.27, *p* = 0.30, two cohort studies, 176 women, I^2^ = 72%).

We further conducted subgroup analysis for women with positive ANA and positive APL and found that the ANA-positive subgroup favored glucocorticoid therapy (RR 2.54, 95% CI 1.09–5.89, *p* = 0.03, three cohort studies, 213 women, *I*^2^ = 51%), while the APL-positive subgroup did not (RR 2.73, 95% CI 0.41–18.27, *p* = 0.30, two cohort studies, 176 women, *I*^2^ = 72%; Figure 3).

#### Live Birth Rate per Couple

**FIGURE 4:**
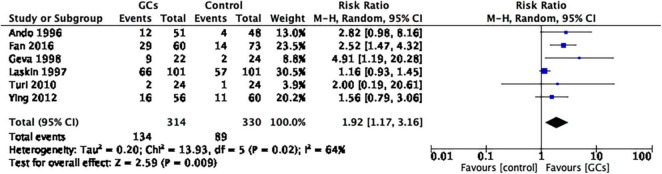
Forrest plot for comparison: glucocorticoids versus no glucocorticoids/placebo, outcome: II. I Live birth rate per couple. A total of seven studies provided the live birth rate by evaluating the effect of glucocorticoid administration. The meta-analysis concluded that it favors peri-implantation glucocorticoids compared to no glucocorticoids (RR 1.92, 95% CI 1.17–3.16, *p* = 0.009, six cohort studies, 644 women, I^2^ = 64%).

A total of six studies evaluated the live birth rate by evaluating the effect of glucocorticoid administration, of which two studies supported glucocorticoid use and four studies showed no significant difference between glucocorticoid and control groups. The meta-analysis concluded that it favored peri-implantation glucocorticoids compared to no glucocorticoids (RR 1.92, 95% CI 1.17–3.16, *p* = 0.009, six cohort studies, 644 women, *I*^2^ = 64%; Figure 4).

**FIGURE 5:**
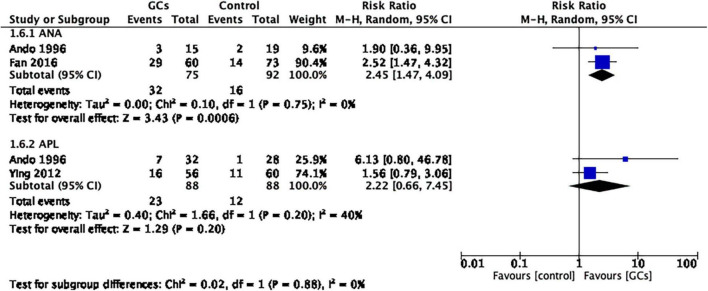
Forest plot for comparison: glucocorticoids versus no glucocorticoids/placebo, outcome: II. II Live birth rate per couple in subgroups by autoantibody. Glucocorticoids improved the live birth rate in women with positive ANA (RR 2.45, 95% CI 1.47–4.09, *p* = 0.0006, two cohort studies, 167 women, I^2^ = 0%). However, in women with positive APL, the result remained controversial (RR 2.22, 95% CI 0.66–7.45, *p* = 0.20, two cohort studies, 176 women, I^2^ = 40%).

We then conducted a subgroup analysis based on autoantibodies. Glucocorticoids improved the live birth rate in women with positive ANA (RR 2.45, 95% CI 1.47–4.09, *p* = 0.0006, two cohort studies, 167 women, *I*^2^ = 0%, Figure 5). However, in women with positive APL, the result remained controversial (RR 2.22, 95% CI 0.66–7.45, *p* = 0.20, two cohort studies, 176 women, *I*^2^ = 40%; Figure 5).

**FIGURE 6:**
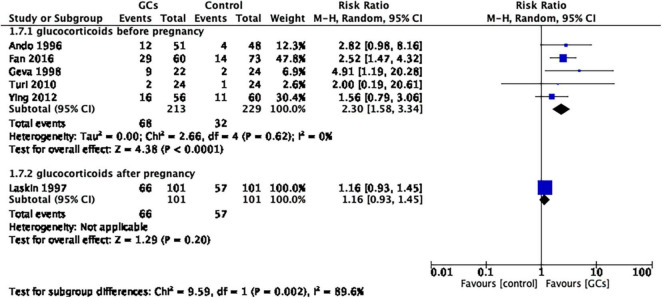
Forrest plot for comparison: glucocorticoids versus no glucocorticoids/placebo, outcome: II. III Live birth rate per couple in subgroups by the time of treatment. Five studies initiated glucocorticoid treatment before confirmation of pregnancy, and one study initiated glucocorticoid treatment after confirmation of pregnancy. The former favors glucocorticoid use (RR 2.30, 95% CI 1.58–3.34, *p* < 0.0001, five cohort studies, 442 women, I^2^ = 0%), while the latter does not (RR 1.16, 95% CI 0.93–1.45, *p* = 0.20, one cohort study, 202 women).

To further understand the effect of glucocorticoids, we divided the studies into two subgroups according to the initial time of treatment. A total of five studies initiated glucocorticoid treatment before confirmation of pregnancy, and one study initiated glucocorticoid treatment after confirmation of pregnancy. The former favored glucocorticoid use (RR 2.30, 95% CI 1.58–3.34, *p* < 0.0001, five cohort studies, 442 women, *I*^2^ = 0%; Figure 6), while the latter did not (RR 1.16, 95% CI 0.93–1.45, *p* = 0.20, one cohort study, 202 women; Figure 6).

#### Miscarriage Rate per Pregnancy

**FIGURE 7:**
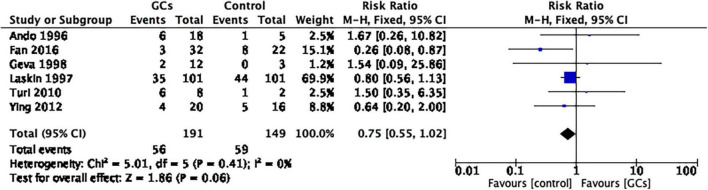
Forrest plot for comparison: glucocorticoids versus no glucocorticoids/placebo, outcome: III. I Miscarriage rate per clinical pregnancy. The use of peri-implantation glucocorticoids on the miscarriage rate per couple showed no statistical significance compared to no glucocorticoids (RR 0.75, 95% CI 0.55–1.02, *p* = 0.06, six cohort studies, 340 women, I^2^ = 0%).

The use of peri-implantation glucocorticoids on the miscarriage rate per couple showed no statistical significance compared to no glucocorticoids (RR 0.75, 95% CI 0.55–1.02, *p* = 0.06, six cohort studies, 340 women, *I*^2^ = 0%; Figure 7).

**FIGURE 8:**
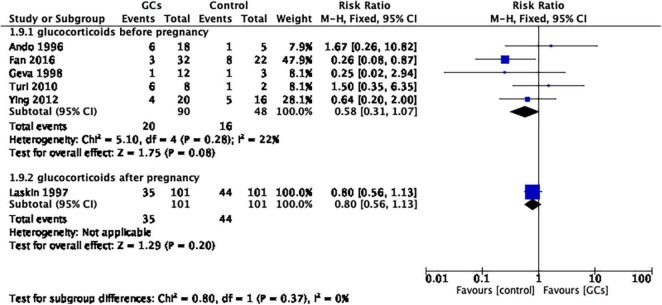
Forest plot for comparison: glucocorticoids versus no glucocorticoids/placebo, outcome: III. II Miscarriage rate per clinical pregnancy in subgroups by the time of treatment. Six studies were divided into two subgroups according to initial treatment time, and the same result was shown for both pre-pregnancy glucocorticoid use and post-pregnancy glucocorticoid use (glucocorticoids before pregnancy: RR 0.58, 95% CI 0.31–1.07, *p* = 0.08, five cohort studies, 138 women, I^2^ = 22%; glucocorticoids after pregnancy: RR 0.80, 95% CI 0.56–1.13, *p* = 0.20, one cohort study, 202 women).

After dividing the studies according to initial treatment time, the same result was shown in both the pre-pregnancy glucocorticoid group and the post-pregnancy glucocorticoid group (glucocorticoids before pregnancy: RR 0.58, 95% CI 0.31–1.07, *p* = 0.08, five cohort studies, 138 women, *I*^2^ = 22%; glucocorticoids after pregnancy: RR 0.80, 95% CI 0.56–1.13, *p* = 0.20, one cohort study, 202 women; Figure 8).

## Discussion

There is a lack of consensus in the treatment of infertile women with unexplained positive autoantibodies. To better understand the effect of a commonly used drug, glucocorticoids, in this population, we conducted this systematic review and meta-analysis. Our study included three types of autoantibodies: ANA, APL, and antithyroid antibodies. ANAs are a diverse group of autoantibodies that recognize nuclear macromolecules and their complexes, which can be characterized in many autoimmune diseases, especially rheumatoid diseases (39). APL is a family of autoantibodies recognizing various combinations of phospholipids, and its role in pregnancy loss is evident from *in vitro* and *in vivo* studies (40). Antithyroid antibodies, which include thyroid-stimulating hormone receptor antibodies (TRAbs), thyroid peroxidase antibodies (TPOAbs), and thyroid globulin antibodies (TgAbs), are associated with thyroid autoimmune diseases (41). We included patients with those autoantibodies which could not be diagnosed as any autoimmune disease, which had certain commonalities, that is, autoimmune abnormalities and repeated pregnancy failure. We analyzed the effects of glucocorticoids therapy on those patients, and the major finding of our study is that glucocorticoid use has a positive effect on improving the clinical pregnancy rate and live birth rate in women with unexplained autoantibodies. The clinical pregnancy rate and live birth rate were improved only in ANA-positive women without specific autoimmune disorders but not in those with positive ACLs. In addition, the effect on live birth rate is significant when glucocorticoids are administered before confirmation of pregnancy rather than after pregnancy.

There were four RCTs and two cohort studies providing data on the clinical pregnancy rate, in which four independent studies favored glucocorticoid use. Our systematic review and meta-analysis based on the current studies confirmed the efficiency of glucocorticoid administration in improving the clinical pregnancy rate in ANA but not APL-positive women, which implies the different pathogenetic roles of diverse autoantibodies in establishing or maintaining pregnancy. However, for subgroups with APL, we found that data extracted from two studies were heterogeneous, and the sample size was small. Thus, examining whether glucocorticoid use can improve the clinical pregnancy rate in APL-positive women needs further research.

The analysis based on five studies providing live birth rates shows the same results: glucocorticoid use has a positive effect in increasing live birth rate only in ANA-positive patients but not in APL. These results indicate that patients with different autoantibodies may also have different immunologic characteristics and clinical outcomes, thus showing different responses to glucocorticoids.

An additional subgroup analysis was done to help determine the best time to start glucocorticoid therapy and showed that preconception use of glucocorticoids improved the live birth rate, while post-conception glucocorticoid administration did not. A meta-analysis conducted by Dan et al. showed that women experiencing unexplained recurrent miscarriage benefit significantly from prednisolone treatment after confirmation of pregnancy in terms of an increased live birth rate compared with placebo (42). However, the population in the studies included in their meta-analysis was different from ours. They used high uterine natural killer cell density (> 5%), a test that is not popular in daily practice, as inclusion criteria, and a known cause for recurrent miscarriage as exclusion criteria, but no mention of autoantibodies (43, 44). Therefore, the difference in the target population of the two studies may underlie the inconsistency in terms of the efficacy of glucocorticoid treatment. Furthermore, no effect of post-conception glucocorticoid treatment may also indicate a risk of fetal exposure to exogenous glucocorticoids. A prospective controlled study collected and followed 311 pregnancies with systemic use of glucocorticoids in the first trimester. Higher rates of miscarriage (11.5% versus 7.0%, *p* = 0.013) and preterm birth (22.7% versus 10.8%, *p* < 0.001) were observed in the glucocorticoid-exposed group than in the controls (45). To date, prednisolone maintains a Category D rating with the Food and Drug Administration in the United States, indicating that routine administration is not recommended. Therefore, further well-designed studies are needed to determine the best initial time and length of glucocorticoid therapy.

The miscarriage rate was not improved by glucocorticoid administration in either the six studies included or our systematic review and meta-analysis. The same result was obtained from further subgroup analysis concerning the initiation time of glucocorticoid treatment. The miscarriage rate in the subgroup “glucocorticoid administration before pregnancy” in our study was 9.4%, which is comparable to the 13.5% reported by Anderson et al. (46). Only one study was included in the post-conception glucocorticoid administration subgroup, and the miscarriage rate was lower than that in the control group without statistical significance. As discussed before, early fetal exposure to exogenous glucocorticoids may cause a risk of miscarriage. Therefore, identifying the optimal indication for glucocorticoid use after confirmation of pregnancy warrants further well-designed studies with large sample sizes.

Our study has several limitations that must be considered regarding the interpretation of the data. First, as mentioned before, our meta-analysis included only seven studies due to a lack of related studies. And different nature of the diseases were included in this study due to the patients with different autobody profiles. However, because the question was so important and the sample size was small in each study and could not give clinicians effective guidance, it was necessary to perform a meta-analysis and systematic review to help us obtain more comprehensive information. Although several borderline effects need to be confirmed by larger sample size, our study still provides preliminary information that is important for further studies. Second, the dosage, type, and therapeutic course of glucocorticoids among the included trials were of great discrepancy. Using a placebo is also inconsistent between studies. Third, the type and dose of adjuvant therapy vary in the included studies, which may affect the interpretation of the effect of GCs therapy, and the effect of these therapies, such as oral aspirin, on changing pregnancy outcomes is not clear (47–49). The effect of glucocorticoid use in women with unexplained positive autoantibodies needs further high-quality investigations to confirm. To gain a better understanding of this therapy, well-designed prospective, randomized, controlled clinical trials must be proposed.

Based on our findings, the recommendations for future study design are listed here. First, glucocorticoids should not be long-acting glucocorticoids, including dexamethasone or betamethasone, because they can pass through the placenta and cause adverse effects such as fetal malformation. Second, 10 mg/d prednisone/prednisolone or less is possibly enough to have a positive effect. Third, a therapeutic course covering 1–3 months before confirmation of pregnancy or controlled ovarian hyperstimulation is recommended, although the effect of post-conception glucocorticoid therapy initiation needs further understanding. Adjuvant therapy such as anticoagulants can be added but should be comparable in both the control and glucocorticoid groups. Fourth, patients should be divided into different subgroups according to their type of autoantibodies.

## Conclusion

Our systematic review and meta-analysis report improvement in the clinical pregnancy rate and live birth rate of glucocorticoid use in women with unexplained positive autoantibodies. Further investigation is required to ascertain its efficacy.

## Data Availability Statement

The original contributions presented in the study are included in the article/Supplementary Material, further inquiries can be directed to the corresponding author/s.

## Author Contributions

TL, YY, RM, and QL conceived and designed the study. TL and YY drafted the manuscript, performed the literature search and data extraction, and assessed the risk of bias and overall quality of evidence. RM revised the manuscript and resolved the disagreement between TL and YY. TL, YY, and HL performed and interpreted the data analysis. All authors read and approved the final manuscript.

## Conflict of Interest

The authors declare that the research was conducted in the absence of any commercial or financial relationships that could be construed as a potential conflict of interest.

## Publisher’s Note

All claims expressed in this article are solely those of the authors and do not necessarily represent those of their affiliated organizations, or those of the publisher, the editors and the reviewers. Any product that may be evaluated in this article, or claim that may be made by its manufacturer, is not guaranteed or endorsed by the publisher.

## Supplementary Material

Supplementary Table 1 Cohort studies excluded and reasons for exclusion.

Supplementary Table 2 Results of bias evaluation of RCT.

Supplementary Table 3 Results of bias evaluation of the retrospective cohort study.
