# Supplementary material for: Glucocorticoids Improve the Pregnancy Rate and Outcome in Women With Unexplained Positive Autoantibodies: A Systematic Review and Meta-Analysis
Source: Front Med (Lausanne). 2022 May 11;9:819406. doi: 10.3389/fmed.2022.819406 (PMC9131042; doi:10.3389/fmed.2022.819406)
Supplement: Supplementary Table 1 — Cohort studies excluded and reasons for exclusion. [file Data_Sheet_1.docx]

Suppl. table 1

| **Study** | **Reason for exclusion** |
| --- | --- |
| **Ordi 1989^[11]^** | Subjects diagnosed immune disease were included |
| **Hasegawa 1992^[14]^** | Subjects diagnosed immune disease were included |
| **Haswgawa 1998^[15]^** | Subjects diagnosed immune disease were included |
| **Vaquero 2001^[12]^** | Subjects diagnosed antiphospholipid syndrome were included |
| **Moradan 2009^[16]^** | Whether the subjects have autoantibody or not wasn’t mentioned |
| **Siristatidis 2013^[17]^** | Whether the subjects have autoantibody or not wasn’t mentioned |
| **Ou 2017^[18]^** | Whether the subjects have autoantibody or not wasn’t mentioned |
| **Ye 2017^[13]^** | Subjects diagnosed antiphospholipid syndrome were included |
| **Siristatidis 2018^[19]^** | Whether the subjects have autoantibody or not wasn’t mentioned |
| **Moffitt 1995^[20]^** | Whether the subjects have autoantibody or not wasn’t mentioned |
| **Geva 2000^[10]^** | The data of some subjects were same as their previously study and the others have no comparison. |
| **Ubaldi 2002^[21]^** | Whether the subjects have autoantibody or not wasn’t mentioned |
| **Stern 2003^[30]^** | GCs were not used |
| **Duvan 2006^[22]^** | Whether the subjects have autoantibody or not wasn’t mentioned |
| **Fawzy 2008^[23]^** | Whether the subjects have autoantibody or not wasn’t mentioned |
| **Kilic 2008^[24]^** | Whether the subjects have normal thyroid function or not wasn’t mentioned |
| **Revelli 2008^[25]^** | Whether the subjects have autoantibody or not wasn’t mentioned |
| **Tang 2013^[26]^** | Whether the subjects have autoantibody or not wasn’t mentioned |
| **Fawzy 2014^[27]^** | Whether the subjects have autoantibody or not wasn’t mentioned |
| **Gomaa 2014^[28]^** | Whether the subjects have autoantibody or not wasn’t mentioned |
| **Litwicka 2015^[29]^** | Whether the subjects have normal thyroid function or not wasn’t mentioned |

Suppl. table 2

| **Study** | **Bias** | **Authors’ judgement** | **Support for judgement** |
| --- | --- | --- | --- |
| **Ando, 1996^[31]^** | Random sequence generation | Unclear risk | Stated as randomized but no further details |
|  | Allocation concealment | Unclear risk | Not reported |
|  | Blinding of participants and personnel | High risk | No blinding |
|  | Incomplete data | High risk | Control woman have undergone a mean number of cycles of 1.6, versus 1.26 for women using GCs, which is significantly different. Likely direction of bias: towards a lack of effect. |
|  | selective reporting | Low risk | All outcomes were reported which were planed according to the methods. |
|  | Other bias | Low risk | No other potential sources of bias identified. |
| **Fan,2016^[35]^** | Random sequence generation | Low risk | computer-generated randomization |
|  | Allocation concealment | Low risk | computer-generated randomization |
|  | Blinding of participants and personnel | High risk | No blinding |
|  | Incomplete data | Low risk | Number of drop outs and cancelled cycles is low. |
|  | selective reporting | Low risk | All outcomes were reported which were planed according to the methods. |
|  | Other bias | Low risk | No other potential sources of bias identified. |
| **Geva,1998^[32]^** | Random sequence generation | Unclear risk | Stated as randomized but no further details |
|  | Allocation concealment | Unclear risk | Not reported |
|  | Blinding of participants and personnel | High risk | No blinding |
|  | Incomplete data | Low risk | Number of drop outs and cancelled cycles is low. |
|  | selective reporting | Low risk | All outcomes were reported which were planed according to the methods. |
|  | Other bias | Low risk | No other potential sources of bias identified. |
| **Laskin,1997^[33]^** | Random sequence generation | Unclear risk | Stated as randomized but no further details |
|  | Allocation concealment | Unclear risk | Not reported |
|  | Blinding of participants and personnel | High risk | No blinding |
|  | Incomplete data | High risk | Number of drop outs is high. |
|  | selective reporting | Low risk | All outcomes were reported which were planed according to the methods. |
|  | Other bias | Low risk | No other potential sources of bias identified. |
| **Turi,2010^[37]^** | Random sequence generation | Low risk | randomized, computerized random number generator |
|  | Allocation concealment | Low risk | opaque sealed envelope |
|  | Blinding of participants and personnel | Low risk | Double blinded. |
|  | Incomplete data | Low risk | Number of drop outs is low. |
|  | selective reporting | Low risk | All outcomes were reported which were planed according to the methods. |
|  | Other bias | Low risk | No other potential sources of bias identified. |

Suppl. table 3

| Study | Bias | Evaluation |
| --- | --- | --- |
| Ying 2012^[36]^ | Representativeness of the exposed cohort | truly representative of the average women with unexplained positive autoantibody in the community |
|  | Selection of the non exposed cohort | drawn from the same community as the exposed cohort |
|  | Ascertainment of exposure | secure record |
|  | Demonstration that outcome of interest was not present at start of study | yes |
|  | Comparability of cohorts on the basis of the design or analysis | study controls for the type of autoantibody |
|  | Assessment of outcome | not mentioned |
|  | Was followed-up long enough for outcomes to occur | yes |
|  | Adequacy of follow up of cohorts | complete follow up |
| Zhu,2013^[34]^ | Representativeness of the exposed cohort | truly representative of the average women with unexplained positive autoantibody in the community |
|  | Selection of the non exposed cohort | drawn from the same community as the exposed cohort |
|  | Ascertainment of exposure | secure record |
|  | Demonstration that outcome of interest was not present at start of study | yes |
|  | Comparability of cohorts on the basis of the design or analysis | study controls for the type of autoantibody |
|  | Assessment of outcome | not mentioned |
|  | Was followed-up long enough for outcomes to occur | yes |
|  | Adequacy of follow up of cohorts | complete follow up |
